# *Kvasir-Capsule*, a video capsule endoscopy dataset

**DOI:** 10.1038/s41597-021-00920-z

**Published:** 2021-05-27

**Authors:** Pia H. Smedsrud, Vajira Thambawita, Steven A. Hicks, Henrik Gjestang, Oda Olsen Nedrejord, Espen Næss, Hanna Borgli, Debesh Jha, Tor Jan Derek Berstad, Sigrun L. Eskeland, Mathias Lux, Håvard Espeland, Andreas Petlund, Duc Tien Dang Nguyen, Enrique Garcia-Ceja, Dag Johansen, Peter T. Schmidt, Ervin Toth, Hugo L. Hammer, Thomas de Lange, Michael A. Riegler, Pål Halvorsen

**Affiliations:** 1 SimulaMet, Oslo, Norway; 2 Oslo Metropolitan University, Oslo, Norway; 3 University of Oslo, Oslo, Norway; 4 Department of Medical Research, Bærum Hospital, Gjettum, Norway; 5 University of Bergen, Bergen, Norway; 6 Augere Medical AS, Oslo, Norway; 7 UIT The Arctic University of Norway, Tromsø, Norway; 8 Karolinska Institutet, Department of Medicine, Solna, Sweden; 9 Ersta Hospital, Department of Medicine, Stockholm, Sweden; 10 Klagenfurt University, Wörthersee, Austria; 11 Medical Department, Sahlgrenska University Hospital-Mölndal Hospital, Göteborg, Sweden; 12 Department of Molecular and Clinical Medicine, Sahlgrenska Academy, University of Gothenburg, Göteborg, Sweden; 13 SINTEF Digital, Oslo, Norway; 14 Department of Gastroenterology, Skåne University Hospital, Malmö Lund University, Malmö, Sweden

**Keywords:** Gastrointestinal bleeding, Pathology

## Abstract

Artificial intelligence (AI) is predicted to have profound effects on the future of video capsule endoscopy (VCE) technology. The potential lies in improving anomaly detection while reducing manual labour. Existing work demonstrates the promising benefits of AI-based computer-assisted diagnosis systems for VCE. They also show great potential for improvements to achieve even better results. Also, medical data is often sparse and unavailable to the research community, and qualified medical personnel rarely have time for the tedious labelling work. We present *Kvasir-Capsule*, a large VCE dataset collected from examinations at a Norwegian Hospital. *Kvasir-Capsule* consists of 117 videos which can be used to extract a total of 4,741,504 image frames. We have labelled and medically verified 47,238 frames with a bounding box around findings from 14 different classes. In addition to these labelled images, there are 4,694,266 unlabelled frames included in the dataset. The *Kvasir-Capsule* dataset can play a valuable role in developing better algorithms in order to reach true potential of VCE technology.

## Abstract (summary)

**Table:** 

Measurement(s)	Gastrointestinal Tract • gastrointestinal system disease
Technology Type(s)	Capsule Endoscope • visual assessment of *in vivo* video recording
Sample Characteristic - Organism	Homo sapiens
Sample Characteristic - Environment	alimentary part of gastrointestinal system

Machine-accessible metadata file describing the reported data: 10.6084/m9.figshare.14178905

## Background & Summary

The small bowel constitutes the gastrointestinal (GI) tract’s mid-part, situated between the stomach and the large bowel. It is three to four meters long and has a surface of about 30 m^2^, including the villi’s surface. As part of the digestive system, it plays a crucial role in absorbing nutrients^1^. Therefore, disorders in the small bowel may cause severe growth retardation in children and nutrient deficiencies in children and adults^1^. This organ may be affected by chronic diseases, like Crohn’s disease, coeliac disease, and angiectasias, or malignant diseases like lymphoma and adenocarcinoma^2,3^. These diseases may represent a substantial health challenge for both the patients and the society, and a thorough examination of the lumen is frequently necessary to diagnose and treat them^4^. However, due to its anatomical location, the small bowel is less accessible for inspection by flexible endoscopes commonly used for the upper GI tract and the large bowel. Since early 2000, video capsule endoscopy (VCE)^5^ has been used, usually as a complementary test for patients with GI bleeding^4^. A VCE consists of a small capsule containing a wide-angle camera, light sources, batteries, and other electronics. The patient swallows the capsule capturing a video as it moves passively throughout the GI tract. A recorder, carried by the patient or included in the capsule, stores the video before a medical expert examines it after the procedure.

VCE devices exist in various versions and brands such as Given Imaging (Medtronic), Ankon Technologies, Chongqing Science, IntroMedic, CapsoVision, and Olympus. The frame rate typically varies between 1 and 30 frames per second, capturing in total between 50 and 100 thousand frames, with pixel-resolutions in the range of 256 × 256 to 512 × 512. Some of the vendors have software to remove duplicated frames due to slow movement. However, a large number of frames need to be analysed by a medical expert, resulting in a tedious and error-prone operation. In the related area of colonoscopy, operator variation and detection performance are reported problems^6–8^ resulting in high miss rates^9^. In VCE analysis, essential findings are missed due to lack of concentration, insufficient experience and knowledge^10–12^. Furthermore, physicians may have trouble handling the associated technology, and infrequent VCE use leads to lack of confidence^13^, resulting in inter-observer and intra-observer variations in the assessments^12^.

The technical developments for automated image and video analysis have sky-rocketed, and multimedia solutions in medicine show tremendous potential^14,15^. An increasing number of promising machine learning solutions are being developed for automated diagnosis of colonoscopies^16–23^ using open datasets^24–27^. Regarding automated VCE data analyses, machine learning approaches also produce promising results regarding detection and classification rates^28–35^. Machine learning, or artificial intelligence (AI) in general, is likely to have profound effects on the VCE technology’s future, not only for improving variation and detection rates but also for estimating the capsule’s localisation^13,36^.

Regardless of promising initial results, there is room for improvements in detection rate, reduced manual labour, and AI explainability. Large amounts of data are needed^37,38^, particularly annotated data^35^, and access to these data are often scarce^39^. As shown in Table 1, very few, small VCE datasets are made publicly available, and several have become unavailable. We have previously published the HyperKvasir dataset^27^. Nevertheless, this and similar datasets containing images from *colonoscopies* and *esophagogastroscopies* are not applicable because they do not depict the small bowel, characterised by the intestinal villi displaying a different surface than the rest of the bowel. Also, the image resolution and the frame rate of VCEs are much lower. The small bowel is not air inflated during a VCE examination, as is the case with traditional colonoscopies. Different optics are also used, and the movement of the capsule is uncontrolled in contrast to flexible endoscopes used during manual examinations.

**Table 1:** An overview of existing VCE datasets from the GI tract.

Dataset	Findings	Size	Availability
KID^54^	Angiectasia, bleeding, inflammations, polyps	2,371 images + 47 videos	open academic^•^
GIANA 2017^55^	Angiectasia^†^	600 images	by request
GIANA2018^56,57^	Polyps and small bowel lesions^†^	8262 images + 38 videos	by request
CAD-CAP^58,59^	Normal frames, fresh blood, vascular lesion, ulcerative and inflammatory lesions	25,000 images	by request^◇^
Gastrolab^60^	Crohns diseases, small bowel (video)+ GI lesions	Few hundred images and videos	open academic^•^

^†^Including ground truth segmentation masks. ^•^Not available anymore.

^◇^The Computer-Assisted Diagnosis for CAPsule endoscopy (CAD-CAP) Database - used for the angiectasia detection.

Therefore, we present a large VCE dataset, called *Kvasir-Capsule*, consisting of 117 videos with 4,741,504 frames and 14 classes of findings. The dataset contains labelled images and their corresponding full videos, and also unlabelled videos. Recent work in the machine learning community has shown significant improvements regarding sparsely labelled and unlabelled data value. Semi-supervised learning algorithms are successfully applied in different medical image analyses^40,41^ using self-learning^42,43^ and neural graph learning^44^. Finally, we provide a baseline analysis and outline possible future research directions using *Kvasir-Capsule*.

## Methods

**Fig. 1:**
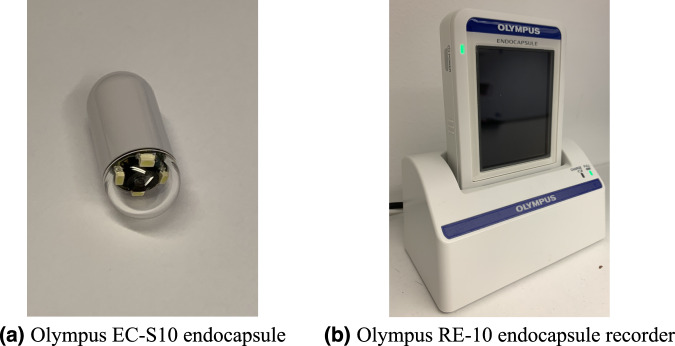
VCE equipment used for data collection.

The VCE videos were collected from consecutive clinical examinations performed at the Department of Medicine, Bærum Hospital, Vestre Viken Hospital Trust in Norway, which provides health care services to 490,000 people, of which about 200,000 are covered by Bærum Hospital. The examinations were conducted between February 2016 and January 2018 using the Olympus Endocapsule 10 System^45^ including the Olympus EC-S10 endocapsule (Fig. 1a) and the Olympus RE-10 endocapsule recorder (Fig. 1b). Originally, the videos were captured at a rate of 2 frames per second, in a resolution of 336 × 336, and encoded using H.264 (MPEG-4 AVC, part 10). The videos were exported in AVI format using the Olympus system’s export tool packaged and encapsulated in the same H.264 format, i.e., the frame formats are the same, but the frame rate specification is changed to 30 fps by the export tool.

Initially, a trained clinician analysed all videos using the Olympus software, selecting thumbnails from lesions and normal findings as part of their clinical work. In spring 2019, all the 117 anonymous videos and thumbnails were exported from a stand-alone workstation using the Olympus software. The Olympus video capsule system has user-friendly functionalities like Omni-selected Mode, skipping images that overlap with previous ones.

All metadata were removed and files renamed with randomly generated file names, before exporting the videos and thumbnails that were shared. Thus, data in the dataset are fully anonymized, as approved by Privacy Data Protection Authority and in accordance with relevant guidelines and regulations of the Regional Committee for Medical and Health Research Ethics - South East Norway. The data has not been pre-processed or augmented in any way apart from this. Subsequently, for clinical analyses of the videos, a central expert reader selected and categorized thumbnails with pathological findings. These thumbnails were traced to their corresponding video segments and the videos were uploaded to a video annotation platform (provided by Augere Medical AS, Norway) for efficient viewing and labelling. Next, three master students labelled and marked the findings with bounding boxes for each frame. The bounding boxes were designed to include the entire lesion and as little as possible of the surrounding mucosa. If the students were unsure about the labelling, the expert reader verified the frames. All labels regarding anatomical structures and normal clean mucosa were then confirmed by one junior medical doctor and the expert reader. Finally, all the annotations were once more verified by the expert reader and subsequently validated by a second expert reader. If the second reviewer disagreed with the annotations, the first reviewer reassessed the images to see whether he then agreed with the second reviewer to get an agreement. After the validation process by the second reviewer there was a disagreement on twenty-six findings in seven examinations; nineteen concerning erroneous terminology of the class lymphoid hyperplasia which was changed to lymphangiectasia. The other seven were related to the interpretation of the finding. After reviewing these findings, the first reviewer agreed with the second one to finally reach a perfect agreement. After this procedure, the video frames were exported as images. Hence, a total of four medical persons have selected, analysed and verified the data, and a total of 47,238 frames are labelled.

The Norwegian Privacy Data Protection Authority approved the export of anonymous images for the creation of the database, without consent from participants. It was exempted from approval from the Regional Committee for Medical and Health Research Ethics - South East Norway. Since the data is anonymised and all metadata removed, the dataset is publicly shareable based on Norwegian and General Data Protection Regulation (GDPR) laws.

## Data Records

**Fig. 2:**
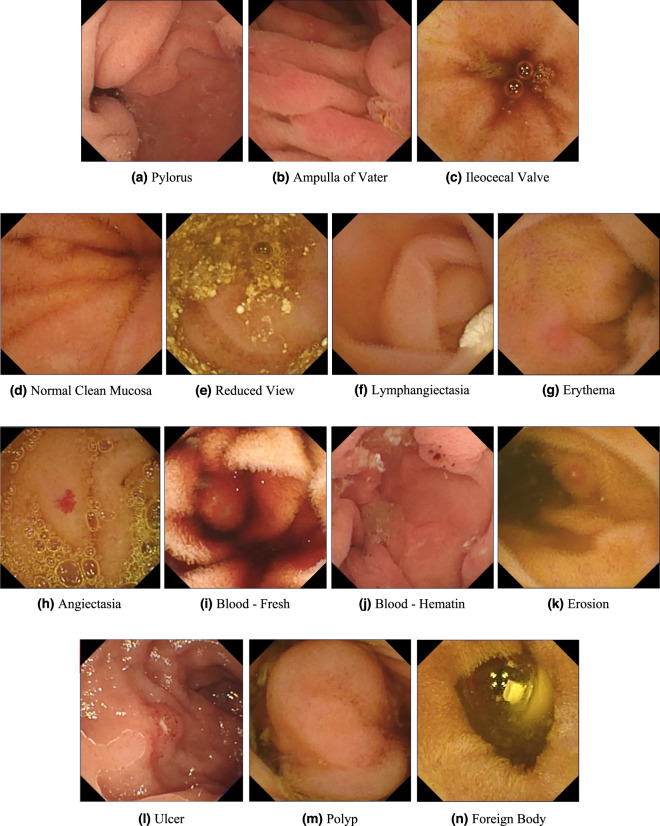
Image examples of the various labelled classes for images. Images (**a**) to (**c**) are from the *Anatomy* category, and images (**d**) to (**n**) are from the *Luminal findings category*.

The *Kvasir-Capsule* dataset is available from the Open Science Framework (OSF)^46^. Table 2 gives an overview of all data records in the dataset. In total, the dataset consists of 4,741,621 main data records, i.e., 47,238 images with labels and bounding box masks, the 43 corresponding labelled videos (the videos from which the images are extracted), and 74 unlabelled videos (from which labelled images have not been extracted). 4,694,266 unlabelled images can further be extracted from all the videos combined. All the various labelled classes are shown in Fig. 2. The dataset has a total size of circa 89 GB. Note that the unlabelled images are not extracted and included in the uploaded data due to unnecessary duplication of data, but can easily be extracted from the videos.

**Table 2:** Overview of the data records in the *Kvasir-Capsule* dataset.

Data Record	# Files
Labelled images	47,238
Labelled videos	43
Unlabelled images	4,694,266
Unlabelled videos	74

The dataset is stored according to the data records listed above, and described in more detail below. We have a “labelled images” catalogue which contains archive files of each labelled class of images. We have a “labelled videos” catalogue which contains all the videos where we have annotated findings from, and an “unlabelled videos” catalogue containing the videos that are not annotated.

### Labelled images

**Fig. 3:**
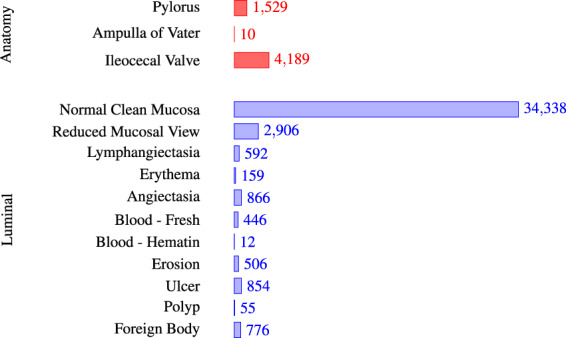
The number of images in the various Kvasir-Capsule labelled image classes.

**Fig. 4:**
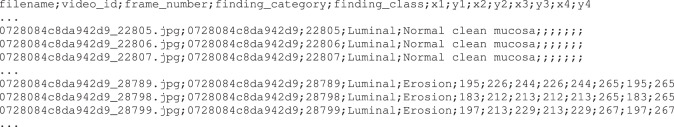
Samples from the *metadata.csv* CSV file.

In total, the dataset contains 47,238 labelled images stored using the PNG format, where Fig. 3 shows the 14 different classes representing the labelled images and the number of images in each class. The provided *metadata.csv* comma-separated value (CSV) file gives the mapping between file name, the labelling for the image, the corresponding video, and the video frame number. Moreover, the CSV file gives information about the bounding box outlining the finding. Some samples are given in Fig. 4 where the first line gives the type of each element in the lines below. This means that the file *filename* of the labelled image which is the frame *frame_number* extracted from the *video_id* video. Moreover, the finding is from the category *finding_category* and class *finding_class*. Finally, the four *x*_*i*_, *y*_*i*_ pairs are the four pixel coordinates for the bounding box, e.g., in the first three lines they are empty, meaning that there is no finding with a bounding box in this labelled image. There is one line in the file per each labelled image.

We defined two main categories of findings, namely anatomy and luminal findings. Each category, their classes and belonging images are stored in their corresponding folder. As observed in Fig. 3, the number of images per class is not balanced. This is a global challenge in the medical field because some findings are more common than others, which adds a challenge for researchers since methods applied to the data should also be able to learn from a small amount of training data.

### Categories of findings

We have organised the dataset in two main categories with their corresponding classes according to the World Endoscopy Association Minimal Standard Terminology version 3.0 (MST 3.0), though we have not included the subcategories or intermediate level to simplify the dataset^47^.

#### Anatomy

The category of *Anatomy* contains anatomical landmarks characterising the GI tract. These landmarks may be used for orientation during endoscopic procedures. However, for small bowel VCE their role is to verify the passage of the capsule trough the entire small bowel to confirm a complete examination. We have labelled three anatomical landmarks, the first two delineate the upper (proximal) and lower (distal) end of the small bowel, respectively. The **pylorus** is the anatomical junction between the stomach and small bowel and is a sphincter (circular muscle) regulating the emptying of the stomach into the duodenum. The **ileocecal valve** marks the transition from the small bowel to the large bowel and is a valve preventing reflux of colonic contents, stool, back into the small bowel. The third one, the **ampulla of Vater**, is the junction between the duodenum and the gall duct.

#### Luminal findings

Endoscopic examinations may detect various *luminal findings*, this include the subcategories content of the bowel lumen, the aspect of the mucosa and mucosal lesions (pathological findings) that could be either flat, elevated or excavated. These subcategories are not shown in the dataset. Normally, the small bowel contains only a certain amount of yellow or brown liquid considered as clean mucosa. However, larger amounts of content may preclude a complete visualisation of the mucosa crucial to verify normal mucosa and detection of all pathological(abnormal) findings. For the lumen content assessment, we have labelled five classes. **Normal clean mucosa** depicts clean small bowel with no or small amount of fluid and mucosa with healthy villi and no pathological findings. This class can also double as a “normal” class versus the pathological luminal finding class (see below). The class **reduced mucosal view** shows small bowel content reducing the view of the mucosa, like stool or bubbles. However, lesions in the upper GI tract or small bowel may bleed, causing the appearance of **blood - fresh** colouring the liquid red. In cases with minimal bleeding, one may observe small black stripes called **blood - hematin** on the mucosal surface. The **foreign body** class include tablet residue or retained capsules which can also be observed in the lumen.

Abnormalities, called lesions or pathological findings, in the small bowel can be seen as changes to the mucosal surface. Typical mucosal changes sometimes cover larger segments, such as a reddish appearance called erythematous mucosa, is labelled as **erythema**. The mucosal wall can also have different focal lesions. The classes of lesions represented in the *Kvasir-Capsule* dataset are **angiectasia**s; small superficial dilated vessels causing chronic bleeding and subsequently anaemia. It mostly occurs in people with chronic heart and lung diseases^48^. Excavated lesions erode to different extents the surface of the mucosa. Most common are **erosion**s, covered by a tiny fibrin layer, while larger erosions are called **ulcer**s. As an example, Crohn’s disease is a chronic inflammation of the small bowel characterised by ulcers and erosions of the mucosa. It may cause strictures of the lumen, making the absorption and passage of nutrients difficult^49^. **Lymphangiectasia**, which represents dilated lymphoid vessels in the mucosal wall, and **polyp**s, which may be precancerous lesions, are visible as protruding from the mucosal wall.

### Labelled videos

Labelled videos are the full 43 videos from which we extracted the above mentioned labelled image classes. In total, these videos correspond to approximately 19 hours of video and 47,238 labelled video frames. Several segments of each video was labelled, and these segments are what was exported as the labelled images. As previously mentioned, one can find the frame number and video of origin of each extracted image in the CSV-file. Even though we already have extracted the most interesting frames (images) found by the clinicians from these videos, they do contain 1,932,047 non-labelled frames that could be interesting in future research. One could also extract the video sequences around the various findings.

### Unlabelled videos

We also provide 74 videos, which contain approximately 25 hours of video and 2,762,219 video frames, without any labels. As previously mentioned, unlabelled data can still have great value. Sparsely labelled or unlabelled data can be important for recently emerging semi-supervised learning algorithms. These videos are of the same format and quality as the labelled videos, except we do not provide any annotations. This means that users of the dataset can either use medical experts to provide further labels, or use the data in unsupervised or semi-supervised learning approaches.

## Technical Validation

To evaluate the technical quality of *Kvasir-Capsule*, we performed a series of classification experiments. We trained two CNN-based classifiers to classify the labelled data. Both architectures have previously shown excellent performance on classifying GI-related imagery from traditional colonoscopies^50,51^, and should be a good benchmark for VCE-related data. The two algorithms are based on standard CNN architectures, namely DenseNet-161^52^ and ResNet-152^53^. All experiments were performed over two-fold cross-validation using categorical cross-entropy loss with and without class weighting. We also used weighted sampling, which balances the dataset by removing and adding images for each class based on a given set of weights. To ensure a fair and robust evaluation, no video is shared between splits. Thus, the frames used for training were independent from the frames used for validation. This also means that there are frames depicting the same finding in each split which then are related to each other, but no related frames distributed across the splits. The effect should therefore be similar to traditional data augmentation techniques used by many researchers today such as multiple rotations, angles and crops.

**Fig. 5:**
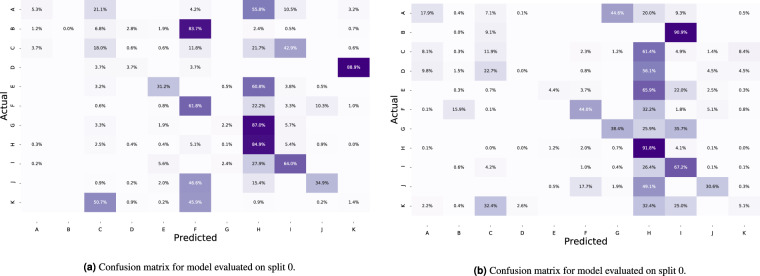
Confusion matrices for the best average MCC value which is from the weighted sampling technique. The labeling of the classes is as follows: (A) Angiectasia; (B) Blood - fresh; (C) Erosion; (D) Erythema; (E) Foreign Body; (F) Ileocecal valve; (G) Lymphangiectasia; (H) Normal clean mucosa; (I) Pylorus; (J) Reduced Mucosal View; (K) Ulcer.

The purpose of these experiments is two-fold. First, we create a baseline for future researchers using the *Kvasir-Capsule* dataset. Second, by using an algorithm that has previously shown good results on classifying GI images, we evaluate how challenging the task of categorizing VCE-related data is. Note that for the classification experiments, we removed the blood - hematin, ampulla of Vater, and polyp classes due to the small number of findings. The results for the two classification algorithms are shown in Table 3 and confusion matrices for the best average MCC value in Fig. 5. We estimated micro-averaged and macro-averaged values for precision, recall and F1-score for each method. The Matthews correlation coefficient (MCC) was calculated using the multi-class generalization, also called the *R*_*K*_. In short, if TP, TN, FP, and FN are the true positives, true negatives, false positives, and false negatives, respectively, these metrics are defined as follows^26^:

**Table 3:** Results for all classification experiments.

Method		Macro average			Micro average			
		Precision	Recall	F1-score	Precision	Recall	F1-score	MCC
Normal CEL	DensNet-161 (fold 0)	0.2165	0.2341	0.1923	0.7375	0.7375	0.7375	0.3707
	DensNet-161 (fold 1)	0.3493	0.3158	0.2996	0.7327	0.7327	0.7327	0.4604
	Avereage	0.2829	0.2749	0.2459	0.7351	0.7351	0.7351	0.4156
	ResNet-152 (fold 0)	0.3302	0.2401	0.1970	0.7203	0.7203	0.7203	0.3520
	ResNet-152 (fold 1)	0.3431	0.2805	0.2789	0.7481	0.7481	0.7481	0.4718
	Average	**0.3367**	0.2603	0.2379	0.7342	0.7342	0.7342	0.4119
Weighted CEL	DensNet-161 (fold 0)	0.2933	0.2939	0.2523	0.7195	0.7195	0.7195	0.3998
	DensNet-161 (fold 1)	0.3163	0.2914	0.2581	0.6991	0.6991	0.6991	0.4054
	Average	0.3048	**0.2927**	0.2552	0.7093	0.7093	0.7093	0.4026
	ResNet-152 (fold 0)	0.2136	0.2872	0.2186	0.6568	0.6568	0.6568	0.3588
	ResNet-152 (fold 1)	0.3033	0.2799	0.2478	0.6890	0.6890	0.6890	0.3966
	Average	0.2585	0.2836	0.2332	0.6729	0.6729	0.6729	0.3777
Weighted sampling	DensNet-161 (fold 0)	0.2525	0.2794	0.2315	0.7332	0.7332	0.7332	0.4111
	DensNet-161 (fold 1)	0.3463	0.2830	0.2806	0.7400	0.7400	0.7400	0.4547
	Average	0.2994	0.2812	**0.2560**	**0.7366**	**0.7366**	**0.7366**	**0.4329**
	ResNet-152 (fold 0)	0.2637	0.2930	0.2334	0.7324	0.7324	0.7324	0.4088
	ResNet-152 (fold 1)	0.3088	0.2619	0.2417	0.7316	0.7316	0.7316	0.4520
	Average	0.2862	0.2774	0.2375	0.7320	0.7320	0.7320	0.4304

Experiments were done with and without weighted cross-entropy loss (CEL) and using a weighted sampling technique. Bold numbers represent the best average value of that column.

### Precision

This metric is also frequently called the *positive predictive value*, and shows the ratio of samples that are correctly identified as positive among the returned samples (the fraction of retrieved samples that are relevant):

$$precision=\frac{TP}{\#\;of\;all\;returned\;samples}=\frac{TP}{TP+FP}$$

### Recall

This metric is also frequently called *sensitivity*, *probability of detection* and *true positive rate*, and it is the ratio of samples that are correctly identified as positive among all existing positive samples:

$$recall=\frac{TP}{\#\;of\;all\;positives}=\frac{TP}{TP+FN}$$

### F1 score (F1)

A measure of a test’s accuracy by calculating the harmonic mean of the precision and recall:

$$F1\;score=2\times \frac{precision\times recall}{precision+recall}=\frac{2TP}{2TP+FP+FN}$$

### Matthews correlation coefficient (MCC)

MCC takes into account true and false positives and negatives, and is a balanced measure even if the classes are of very different sizes. For the multiclass classification generalization, it is often called the *R*_*k*_ statistic. In following equation, *t*_*k*_ is the number of times class *k* actually occurred, *p*_*k*_ is the number of times class *k* was predicted, *c* is the total number of samples correctly predicted, and *s* is the total number of samples:

$$MCC=\frac{c\times s-{\sum }_{k}^{K}\,{p}_{k}\times {t}_{k}}{\sqrt{({s}^{2}-{\sum }_{k}^{K}{p}_{k}^{2})\times ({s}^{2}-{\sum }_{k}^{K}{t}_{k}^{2})}}$$

The micro and macro averages are different ways to average metrics calculated over multiple classes. The macro average is the arithmetic mean of all the scores of different classes, i.e., calculates the metric per class and then calculates the average of these over the number of classes. For example, it is defined for precision as the sum of precision scores for all classes (precicion_1_ + … + precicion_*n*_) divided by the number of classes (*n*). The micro average is not counting class wise first, but looking at the total number of true and false findings. For example, for precision, it is defined as sum of true positives (TP_1_ + … + TP_*n*_) for all the *n* classes divided by the all returned positive predictions (TP_1_ + FP_1_ + … + TP_*n*_ + FP_*n*_).

Considering the results, we experience that classifying VCE data is quite a challenging task. For example, several of the classes are erroneously predicted as **Normal clean mucosa**. On the other hand, the class with the most accurate predictions is also **Normal clean mucosa**, reaching 85% in fold one and 91% in fold two. This is expected as the class comprise approximately 73% of the labelled images. This points out the challenges of making reliable systems as there are multiple aspects to consider, e.g., the resolution of VCE frames are lower compared to gastro- or colonoscopies, and many of the findings are subtle where even clinicians have difficulties differentiating between the classes. As noticed when comparing the images in Fig. 2, several findings are hard to see and easily mixed. For example, erosions can often be mistaken as small residues, and it can be difficult to differentiate normal mucosa from slight erythema. Thus, these results show the potential of AI-based analysis, but also further motivates the need to publish this dataset for more investigations and research into better specific algorithms for VCE data. The code used to conduct all experiments, produce all plots, and the images contained in each split are available on GitHub (https://github.com/simula/kvasir-capsule), i.e., to increase reproducibility and facilitate researches to perform comparable experiments on the *Kvasir-Capsule* dataset.

## Usage Notes

To the best of our knowledge, we have collected the largest and most diverse public available VCE dataset. *Kvasir-Capsule* is made available to enable researchers to develop detection or classification methods of various GI findings using for example computer vision and machine learning approaches. As the labelled findings also include bounding boxes, areas of potential use are analysis, classification, segmentation, and retrieval of images and videos of particular findings or properties. Moreover, the ground truths of various findings by the expert gastroenterologists provide a unique and diverse learning set for future clinicians, i.e., the labelled data can be used for teaching and training in medical education.

The unlabelled data is well suited for semi-supervised and unsupervised machine learning methods, and, if even more ground truth data is needed, the users of the data can have medical experts provide the needed labels. In this respect, recent work has shown remarkable improvements in the area of semi-supervised learning, also successfully applied in medical image analyses^40^. Instead of learning from a large set of annotated data, algorithms learn from sparsely labelled and unlabelled data. Self-learning^42,43^ and neural graph learning^44^ are both examples using unlabelled data in addition to a small amount of labelled data to extract additional information^41–43^. In an area with scarce data, these new algorithms might be the technology needed to make AI truly useful for medical applications.

An important note in general for this type of AI-based detection systems is that one should be careful about how the dataset is split into for example training and test sets in order to avoid having related frames in several of the sets. This will give an unfair effect on the results. Thus, the splits should be completely different, probably even at the level of patients. As described below, an example of such a split in found in our GitHub repository (see below in the Code Availability section).

Currently, there is substantial research in GI image and video analysis. We welcome future contributions such as using the dataset for comparisons and reproducibility of experiments and further encourage publishing and sharing of new data. *Kvasir-Capsule* is licensed under a Creative Commons Attribution 4.0 International (CC BY 4.0) License, which permits use, sharing, adaptation, distribution and reproduction in any medium or format, as long as you give appropriate credit to the original authors and the source.

## Data Availability

In addition to releasing the data, we also publish code used for the baseline experiments. All code and additional data required for the experiments, including our splits into training and test datasets, are available on GitHub via http://www.github.com/simula/kvasir-capsule.
